# Therapeutic potential of nanotechnology-based approaches in osteoarthritis

**DOI:** 10.3389/fphar.2022.920824

**Published:** 2022-08-08

**Authors:** Likang Xiao, Jiarui Cui, Zhuang Sun, Yunke Liu, Jia Zheng, Yonghui Dong

**Affiliations:** ^1^ Department of Orthopedics, Henan Provincial People’s Hospital, Zhengzhou University People’s Hospital, Henan University People’s Hospital, Zhengzhou, China; ^2^ School of Rehabilitation and Health Preservation, Chengdu University of Traditional Chinese Medicine, Chengdu, China

**Keywords:** nanotechnology, nanomedicine, osteoarthritis, drug delivery system, treatment

## Abstract

Osteoarthritis (OA) is a multifactorial disease that affects the entire joint, often resulting in severe pain, disability, psychological distress, and a lower quality of life. Patient self-management is emphasized in OA clinical recommendations. Currently, the clinical treatment of OA mainly focuses on pain relief and the improvement of joint function, with few options for regenerating degenerative cartilage or slowing the progression of OA. Therefore, we first reviewed the current treatment of OA, and then summarized the research advances of nanotechnology in OA treatment, including nano drug delivery systems for small molecule drugs, nucleic acids and proteins, nano-scaffolds for cartilage regeneration, and nanoparticle lubricants. Finally, we discussed the opportunities and potential challenges of nanotechnology in OA treatment.

## 1 Introduction

Osteoarthritis (OA) is a degenerative joint disease characterized by joint pain, tenderness, deformity and dysfunction, which often occurs in weight-bearing joints such as knee and hip. Strain, trauma and deformity can cause cartilage injury and osteophyte hyperplasia, which lead to the occurrence of osteoarthritis, and the incidence rate is increasing year by year, negatively impacting the life quality of OA patients and added a heavy burden to social medical resources. At present, the treatment of OA is relatively complete, but neither drugs nor surgery can yield satisfactory results (Bennell et al., 2014). Since the first proposal by physicists more than 60 years ago, nanotechnology has been widely applied in various fields. Nanotechnology has opened up new ideas for the diagnosis and treatment of many diseases (Bennell et al., 2014). Nanotechnology has showed potential in the treatment of OA in drug delivery, biological scaffolds, genetic engineering, and lubricants. Many achievements have been made in the field of bone tissue engineering with nanotechnology (Bennell et al., 2014). In this paper, we first introduce the current state of OA treatment. Then, by searching the latest related research, the research progress of nanotechnology in the treatment of osteoarthritis was reviewed from the aspects of drug delivery system, gene delivery system, osteocartilage regeneration scaffold, nano lubricant. Finally, we comment on the prospect and possible challenges of nanotechnology in the treatment of OA.

## 2 Current OA treatment

At present, a relatively reasonable step-by-step treatment plan for OA has been basically formed. Patients received graded and targeted therapy according to the etiology, severity, and imaging classification. Early OA can be alleviated through loading reduction (reducing the frequency of sports such as climbing stairs and mountains), oral medication [glucosamine, non-steroidal anti-inflammatory drugs (NSAIDs)], and physical therapy. Patients may need to wear braces or receive intra-articular therapy as the problem worsens. In the late stage of OA, arthroscopic cleaning along with meniscal repair, osteotomy or joint replacement may be required.

### 2.1 Conservative treatment

In the early stage of OA, it is beneficial to spread fundamental joint protection knowledge to patients. They are recommended to engage in joint-friendly activities, such as swimming, walking, and flexion-extension exercises, as well as adjust their lifestyle to lose weight and avoid excessive joint weight-bearing and movement (Bennell et al., 2016). Functional exercise can help patients with moderate to severe OA relieve pain and improve joint function to some extent, but it is ineffective in improving cartilage health and slowing the progression of OA (Bennell et al., 2014).

NSAIDs are commonly used analgesics in clinical OA patients, and the incidence of drug-related adverse effects induced by NSAIDs is much lower than that generated by opioids (Krebs et al., 2018). NSAIDs inhibit cyclooxygenase activity and reduce prostaglandin production, which may induce pain and inflammation. When taking NSAIDs for a long time, attention should be paid to prevent gastric mucosal damage (Langworthy et al., 2010). Although glucosamine and chondroitin can improve cartilage function, evidence from a large sample and multi-center study suggests they have little effect on OA (Felson, 2007). Bisphosphonates can delay joint replacement in OA patients by reducing bone degradation, although more clinical trials are needed to confirm the particular efficacy (Neogi et al., 2018).

Hyaluronic acid (HA) is an inherent component of the human body and has no species specificity. It has the ability to lubricate joints, protect cartilage, and relieve pain. Intra-articular injections of HA can increase the viscoelasticity of synovial fluid while causing less systemic adverse reactions (Zychowicz, 2014). Intra-articular injection of glucocorticoids is recommended for severe OA that is not responsive to NSAIDs. However, compared with physical therapy, intra-articular injections of glucocorticoid have more adverse reactions (Bennell et al., 2014), and long-term use will exacerbate articular cartilage loss. Platelet-rich plasma (PRP), which contains platelets and a variety of growth factors, plays an active role in meniscus and cartilage repair. PRP can be used to treat all stages of OA, particularly early OA, with a superior therapeutic effect (GöRmeli et al., 2017).

### 2.2 Surgical treatment

**FIGURE 1:**
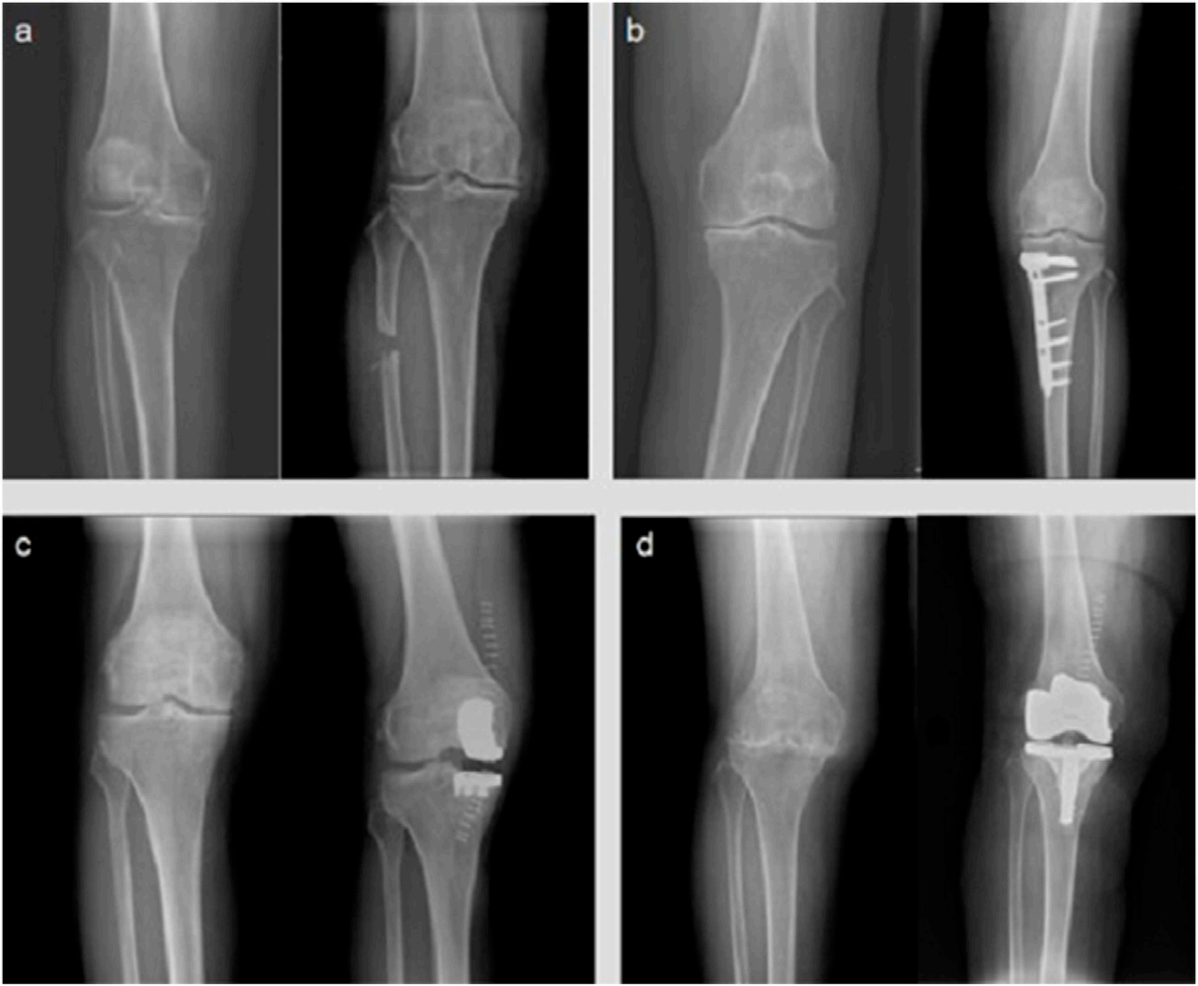
Digital radiographs of knee OA patients. Here is the comparison of the main surgical methods for the treatment of knee osteoarthritis before and after different surgeries (left: before surgery; right: after surgery) operation, each picture shows the digital radiography before and after the operation: **(A)** proximal fibula osteotomy; **(B)** high tibial osteotomy; **(C)** unicompartmental knee arthroplasty; **(D)** total knee arthroplasty.

Surgery is a part of the comprehensive treatment of OA (Figure 1). It can assist in the diagnosis of OA, relieve pain, correct deformity, prevent additional joint degeneration, and improve joint function. Arthroscopy has had a considerable impact on the diagnosis and treatment of joint diseases throughout the last few decades. The basic approaches for treating OA under arthroscopy are loose body removal and joint cleaning. However, arthroscopic treatment of OA, particularly severe OA, does not improve therapeutic outcomes (Kirkley et al., 2008).

Knee OA is frequently coupled with joint deformity, causing stress to be localized in the medial compartment of knee. Varus deformity can be induced by fibular support on the lateral side of the proximal tibia, causing uneven settlement of the medial and lateral parts of the knee joint. On this basis, the proximal fibula osteotomy (PFO) was designed to successfully relieve the pain by reducing the pressure in the medial compartment of the knee joint. PFO has the advantages of simple operation, small trauma, and less adverse reactions, therefore it has a promising clinical future (Baldini et al., 2018; Sugianto et al., 2021). High tibial osteotomy (HTO), which improves knee function by shifting the force line of the knee joint, is also an important part of knee-protection treatment for OA. HTO has a good impact in pain relief, but it is important to understand the surgical indications and arrange the orthopedic degrees effectively, or it will be difficult to produce a suitable effect for OA patients (van Outeren et al., 2017). Another excellent surgical option for single-compartment OA is unicompartmental knee arthroplasty (UKA). UKA, like HTO, requires precise indication prior to surgery (Jeon et al., 2017).

Total joint replacement (TJA) can help patients with advanced OA achieve good clinical outcomes. With the advancement of prosthesis material science and the updating of TJA theory, more and more surgical technologies emerge as time goes on, allowing orthopedists to choose the best surgical plan for different types of OA (Moorthy et al., 2021). Revision of the prosthesis after it has outlived its usefulness, as well as the possibility of infection around the prosthesis, are still unsolved issues. The process of exploring how to solve these problems in the future will be a protracted war (Taunton, 2021).

## 3 Nano drug delivery system in OA treatment

OA is accompanied by a number of microscopic alterations as well as a degeneration of joint function caused by the damage to essential joint components, such as synovium, muscle, ligament, and cartilage. When compared to other drug administration methods, local drug injection can cause them to concentrate in the joint capsule, resulting in a more acceptable therapeutic effect (Larsen et al., 2008). However, the drugs will be gradually cleaned up after entering the joint cavity, and the removal time is related to the size of the drug particles. The short half-life drugs necessitate multiple injections to obtain the desired effect, which obviously increases the risk of infection. To compensate for these flaws, a more advantageous drug delivery system mechanism is urgently required (Jones et al., 2019).

**FIGURE 2:**
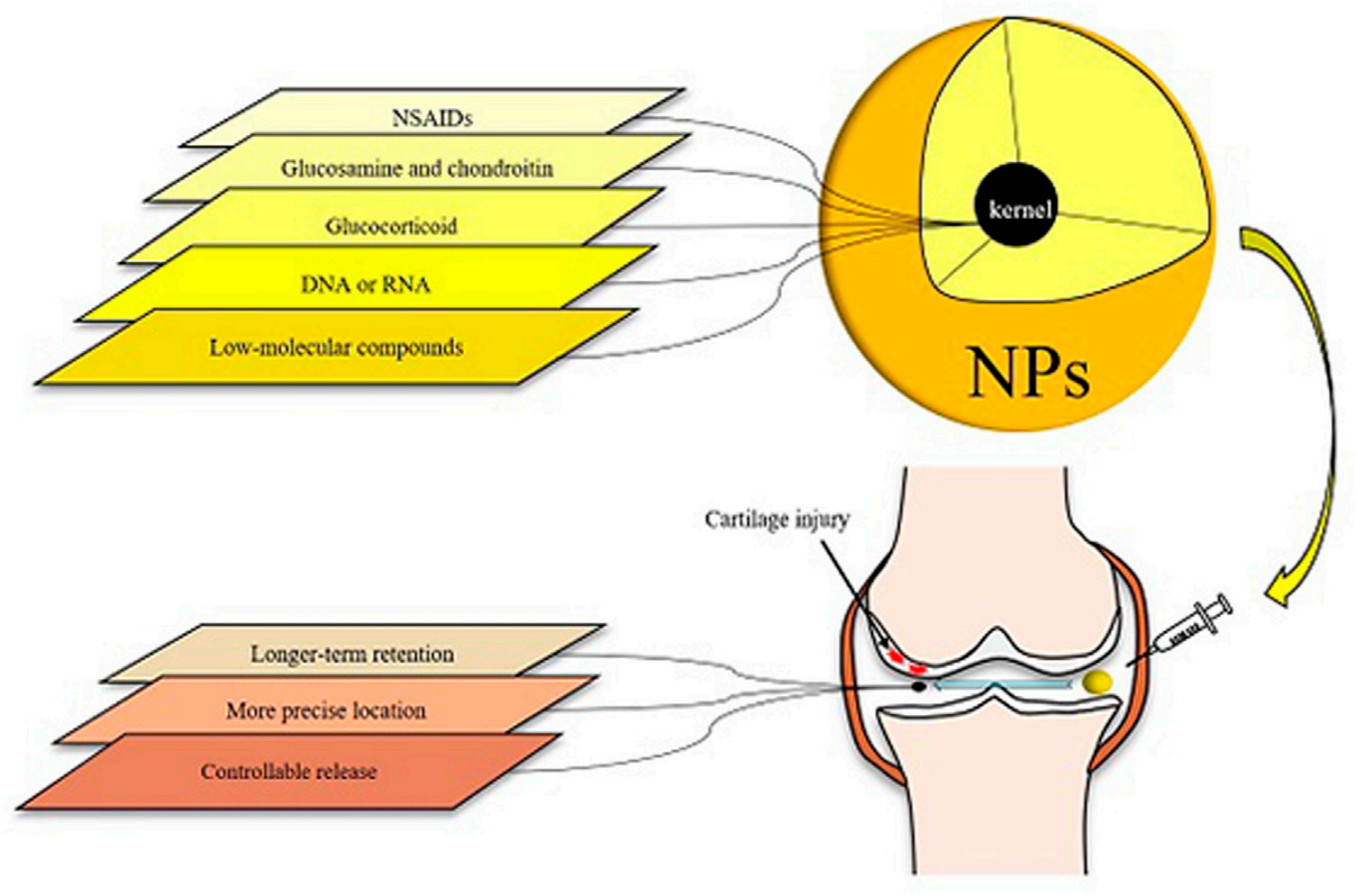
Various types of drugs for osteoarthritis can be loaded in NPs and then introduced into the articular cavity. The specific conjugates assembled with OA targets can mediate the action of NPs to a specific location, thus achieving accurate localization. Nanoscale cladding with the property of evading scavenging allows the drug to act longer. When the carrier has the characteristics of temperature sensitivity or PH sensitivity, the drug can have adjustable release characteristics.

Nanotechnology has been used in a variety of medical sectors, including oncology and cardiology. Nanoparticles (NPs) have good biocompatibility and degradability (Medina et al., 2007; Liang et al., 2021), and have been shown to be capable of carrying several types of medications, such as DNA, peptides and low-molecular compounds. NPs can penetrate through various barriers, and the production of NPs that encapsulate or bind drug molecules can improve the solubility, stability, and absorption capacity of medications, preventing them from being removed prematurely during transportation. The targeted delivery of drug molecules to specific targets has been realized thanks to the advancement of nanotechnology, and NPs-based drug delivery systems may fully meet the requirements of accurately releasing medications to specific targets. This can help drugs perform its potential while also reducing its side effects. In Figure 2, we show the brief structure of NPs and its action process in the joint cavity, and list the advantages of NPs (Figure 2).

### 3.1 Nanoparticle polymers

Natural polymers are biocompatible because their extracellular matrix (ECM) similar to that of human tissue, they can prevent an immune response by modifying cell adhesion, and they disintegrate quickly through natural enzymatic or chemical processes. Natural polymers, on the other hand, have little mechanical strength, making their use in the medical field difficult (Puertas-Bartolomé et al., 2021).

Chitosan is the only naturally occurring basic polysaccharide. Because chitosan has a significant number of amino groups, it can react with the anionic system, generating physical and chemical alterations. Chitosan and glycosaminoglycan have similar structures that can promote cartilage formation. Kartogenin (KGN) is a small molecule that stimulates the differentiation of human bone marrow mesenchymal stem cells into chondrocytes and is injected into the joint cavity with chitosan NPs (KGN conjugated chitosan NPs) to successfully prevent cartilage degeneration (Kang et al., 2014). This is achieved by extending the retention time of the drug. Anti-inflammatory activities of glucocorticoids are excellent. Intra-articular glucocorticoid injection is a frequent treatment for OA, however the potency of drugs in the joint cavity is diminished after passing through many obstacles. The ECM of cartilage has a high metabolic rate, which permits medications to penetrate the cartilage. When dexamethasone is combined with polycationic chitosan [Chitosan-dexamethasone loaded poly (ε-caprolactone) nanofibers], a strong electrostatic effect is generated between the dexamethasone and the ECM of cartilage cells, allowing the dexamethasone to continuously penetrate into the entire layer of cartilage and release active dexamethasone *via* ester bond hydrolysis, greatly increasing the therapeutic effect of dexamethasone on cartilage (Formica et al., 2019).

Aliphatic polyesters such as polylactic acid and polyglycolide, as well as their copolymers have been frequently used in NP engineering because of their biocompatibility and biodegradability. The degradation products of these polymers are lactic acid and glycolic acid, which are two metabolites of the citric acid cycle (Krebs cycle). The emulsion solvent evaporation method was used to make etocoxib-containing NPs from polylactic acid and chitosan hydrochloride as raw materials. It has the characteristics of small particle size and high drug release, and it may be used to treat inflammatory and bone remodeling. The etoricoxib loaded-polylactic acid-chitosan hydrochloride/Captex200/Tween80 NPs (PLA-CS NPs) possessed the smallest particle size and the most sustained drug release by contain the hydrophilic part concentrated on the outer surface, which is dissolved to form a channel, saturated drug solutions will appear in these channels, causing other drug molecules to slowly spread into the surrounding fluid. PLA-CS NPs exhibit good cell compatibility on MC3T3-E1 normal bone cell lines, and may improve alkaline phosphatase activity, as well as the deposition and binding of calcium ions (Salama et al., 2020).

Targeting technology improves the efficiency of intra-articular drug delivery. CD44 (a receptor for HA) expression is higher in articular cartilage of OA patients and mice, and self-assembled HA-NPs [HA/5β-cholanic acid (CA)-NPs] can actively target CD44. Compared with free high molecular weight HA, HA-NPs showed *in vitro* resistance to hyaluronidase digestion and long-term retention within the knee joint (Kang et al., 2021). Self-assembled NPs binding interleukin (IL)-1 receptor antagonist (IL-1RA) can extend the half-life of IL-1RA in rat cartilage (Rey-Rico et al., 2017). Experiments *in vivo* revealed that a cartilage-targeting polymer drug delivery system with a diameter of around 218 nm and formononetin-polyethylene glycol (PEG) (polyethylene glycol-formononetin conjugated nano-sized drug delivery vehicles) might successfully halt the progression of OA. The combination of polyethylene glycol and collagen B-peptide has high cell penetration efficiency, it makes it easier for the drug to penetrate the dense cartilage matrix. (Bennell et al., 2014). Controlling particle size and charge in targeting technology can improve drug retention in the ECM and joint cavity. Amine-terminated polyamidoamine (PAMAM) dendrimers are made up of 64–256 primary cationic amines with dense surface functional groups and terminals functionalized with different molar ratios of PEG to modulate surface charge. After combined with insulin-like growth factor 1, PAMAN-poly (ethylene glycol)-IGF-1 NPs may penetrate through the entire bovine cartilage layer in 2 days, and its residence period in the knee joint of rats increased by 10 times (Geiger et al., 2018).

### 3.2 Stimulate reactive NPs

Only certain environmental circumstances or when stimulation is present cause stimulate reactive NPs to release their formulation. Disease-related changes in local environmental parameters, such as temperature, pH, and oxidative stress, as well as external stimuli like near infrared light and x-ray, are examples of triggers.

Anti-inflammatory medications are more effective in treating OA when they are delivered in a targeted manner, which decreases the therapeutic dose and side effects. Song et al. synthesized temperature-sensitive hollow dextran/poly (N-isopropylacrylamide) NPs by destroying the N, N′-bisacryloylcystamine crosslinked nucleus in imidazolium-based ionic liquids. Dynamic light scattering, scanning electron microscopy, and transmission electron microscopy were used to examine their heat response. The sulfur functionality boosts therapeutic KAFAK peptide loading. In cartilage explants, KAFAK-loaded hollow dextran/poly (N-isopropyl acrylamide) NPs effectively deliver therapeutic peptides to inhibit inflammation (Song et al., 2021). Lachowicz et al. proposed that the condensed polysaccharide NPs could transport and release the hydrophobic drug piroxicam (PIX) into cells in response to temperature changes (Lachowicz et al., 2020). The system is based on the ionic derivatives of natural polysaccharide-curdlan and hydroxypropyl cellulose. Curdlan is treated with trimethylammonium groups to give the anionic derivative of hydroxypropyl cellulose by the introduction of styrenesulfonate groups. As a result of the agglomeration process, from the thermally responsive NPs in which the obtained ionic polysaccharide spontaneously forms a spherical shape in water and the average hydrodynamic diameter is in the range of 250–300nm, the PIX is effectively encapsulated within the NPs. The morphology can be observed by scanning electron microscope and atomic force microscope. The size and surface charge of the produced objects can be changed by adjusting the polycation to polyanion ratio. The release curve of drug from the system could be influenced by changing the temperature (Lachowicz et al., 2020).

To prevent the progression of post-traumatic OA (PTOA), Deloney et al. designed hollow solid thermoreactive NPs (poly (NIPAm-co-AMPS-AAc-BAC) NPs) that was successfully delivered to the joint cavity of rats by intra-articular injection (Deloney et al., 2020). The system takes advantage of the heat-sensitive properties of N-isopropylacrylamide. NPs expand below the critical solution temperature and shrink above the critical solution temperature. Non-crosslinked poly-N-isopropylacrylamide (pNIPAm), which is maintained above the critical solution temperature, forms a hydrophobic core with a shell formed by the polymerization of N-isopropylacrylamide, degradable crosslinker N, N′-bis(acryloyl)cystamine, sulfated 2-acrylamido-2-methyl-1-propanesulfonic acid and acrylic acid. The uncrosslinked pNIPAm core is removed by diffusion produced thermosensitive, degradable NPs with low density or hollow, cores. Compared with solid NPs, hollow NPs increase the drug load, are absorbed into chondrocytes within 24 h and cleared from cells within 6 days, significantly reducing the secretion of pro-inflammatory cytokine IL-6 (Deloney et al., 2020).

The delivery of multiple drugs with different efficacies in a single system can improve the treatment efficiency of diseases, and the accurate and independent control of the release of each drug is an important problem faced by the system. Kang et al. designed thermally responsive polymer nanospheres (F127/COS/KGN_DCF_) to provide simultaneous and independent dual drug delivery in response to temperature changes (Kang et al., 2016). In order to achieve the dual drug release of KGN and diclofenac, KGN is covalently crosslinked to the outside of the nanospheres, and diclofenac is loaded into the inner core. Controlling the release of the two drugs by changing the temperature could inhibit the inflammation response and promote cartilage formation in OA rats (Kang et al., 2016).

The levels of reactive oxygen species (ROS) and reactive nitrogen species (RNS) in chondrocytes are usually low, but they are up regulated in OA patients, and specific inhabitation of the proliferation of activated macrophages and elimination of high ROS secreted by macrophages are important for OA treatment. Most free radical scavengers are less biocompatibility and cytotoxicity. Dopamine NPs have excellent anti-inflammatory and cartilage protection effects by inhibiting intracellular ROS and RNS and promoting antioxidant enzyme activity. Dopamine NPs can be injected intra-articularly and retained at the injection site. Animal experiments have shown that dopamine NPs can reduce the release of inflammatory cytokines and the loss of proteoglycans, thereby slowing cartilage degradation. Dopamine NPs can also enhance autophagy, which is beneficial for OA control (Zhong et al., 2019). Yang et al. used folic acid-modified HA to wrap the surface of physically encapsulated CO-releasing molecule particles [CORM-401/folic acid (FA)/hyaluronic acid (HA)]. and constructed a multifunctional anti-inflammatory drug based on peptide-based dendritic polymer nanogel (Yang et al., 2020). The nano system can effectively enable drugs to enter activated macrophages through specific targeting mediated by FA and HA, thereby depleting ROS in joints, which can effectively inhibit the degradation of articular cartilage and its extracellular matrix, and the system has no toxicity to normal macrophages (Yang et al., 2020).

Near-infrared (NIR) light is an electromagnetic wave between visible light and mid-infrared light with a wavelength of 780–2526 nm. NIR light can penetrate through tissues and has the characteristics of low absorption, less scattering, and less autofluorescence. Zhao et al. proposed that chitosan-modified molybdenum disulfide nanosheets (MoS2/chitosan/dexamethasone) as the response carriers of NIR, loaded with dexamethasone (Dex), which is released after being triggered by photothermal conversion (Zhao Y. et al., 2019). By adjusting the radiation behavior of NIR light, the release of Dex in the joint cavity can be controlled remotely to prolong the storage time of Dex in the joint cavity (Zhao Y. et al., 2019).

### 3.3 Micelles and liposomes

The micelles are made up of amphiphilic polymers with diameters ranging from 20 to 200 nm that self-assemble in aqueous solutions. Hydrophobic medications are wrapped around the core of the micelle, while hydrophilic medications can be used to fix the surface. Micelles are taken up preferentially when polypeptides, antibodies or other targeted ligands bind to them. Polyethylene glycol can protect the micelles from being phagocytosed and is the site for further modification.

Micellar drug delivery systems for the treatment of OA are common. Wu et al. assembled a hydrogen peroxide-sensitive nanomicelle (PLGA-SeSe-mPEG) (Wu et al., 2021). The NPs have uniform size and an obvious core-shell structure. Under the stimulation of H_2_O_2_, the shell layer can be gradually removed, and then Dex and cartilage-derived optogenetic protein-1, in the micelles are released to induce bone marrow mesenchymal stem cells to repair cartilage and inhibit joint inflammation at the same time (Wu et al., 2021). Acidic environment and overexpression of matrix metalloproteinase (MMP)-13 are typical markers of OA. Lan et al. reported a stimulus-responsive nanomicelle (MRC-PPL@PSO) whose drug release is controlled by pH and MMP-13 (Lan et al., 2020). The NPs combines cartilage-specific gene sequence to provide sustained drug release in response to acidic conditions. The high efficiency of this targeted and precise therapy has been confirmed by experiments *in vitro* and *in vivo* (Lan et al., 2020). In the presence of IL-1β and tumor necrosis factor-α, polymer micelle (Copolymer PF68/T908) based on polyethylene oxide and polypropylene oxide can induce gene overexpression in human chondrocytes, enhance the deposition of extracellular matrix components and cell survival level by counteract the specific contribution of major OA-associated inflammatory cytokines in chondrocyte cultures, and effectively reverse the harmful effects of OA cytokines on these processes (Urich et al., 2020).

As well as micelles, liposomes are self-assembled structures. Liposomes have a water core surrounded by bilayer lipids and can easily encapsulate hydrophilic or polar reagents. Liposomes vary in size from 50 to 5000 nm, depending on the composition and formulation, and are commonly used to deliver anticancer agents. The drug-loaded nanostructured liposomes are mixed with hyaluronic acid-liposome-diclofenac/dexamethasone system (HA- Lipo-DIC/DEX), it has the best stability and maintains the effectiveness and encapsulation of drug delivery without loss before it reaches the target organ. The effective working concentration can be reached within 4 h, and the drug release time is at least 168 h without significant toxicity. Sufficient intra-articular injection of the system can effectively reduce the incidence of OA in mice (Chang et al., 2021). A recent clinical trial proposed that the efficacy of diclofenac liposomal gel in the treatment of OA patients was better than that of oral formulations, indicating that the drugs in the liposomes had higher therapeutic potential (Bhatia et al., 2020). Corciulo et al. reported that intra-articular injection of suspension of selective A2A receptor agonist CGS21680 could significantly reduce cartilage damage in mouse OA model (Corciulo et al., 2020). In the OA rat model, the same treatment also improved the swelling of the affected knee joint and cartilage preservation (Corciulo et al., 2020). The liposome system significantly improves the clinical efficacy of the anti-inflammatory drugs, controls drug release, and reduces the occurrence of adverse reactions.

## 4 Gene delivery system

With the development of gene technology, gene therapy has attracted increasing attention, and various therapeutic targets for OA have been found, which provides a partial theoretical basis for the use of NPs to deliver gene therapy. Efficient gene therapy mainly depends on the control highly unstable genetic materials to achieve the therapeutic goals, which requires an efficient and safe delivery system (Dorraj et al., 2017). In response to this, various delivery systems have been developed. Viral vectors have been widely used in current gene therapy experiments, but they have high immunogenicity, high cost and many potential risks. A non-viral delivery system may be a better option and is currently in clinical trials at different stages, such as the development of a solid lipid NP system that delivers p-IL10 to transfect the cornea to treat corneal diseases, and the treatment of cancer based on DOTAP, modified PEG (MPEG)-poly (ϵ-caprolactone) (PCL)-MPEG and FA-MPEG-PCL-PEG-FA self-assembled folic acid modified gene delivery system (Vicente-Pascual et al., 2018; Nie et al., 2021).

Exosomes act as intercellular messengers, can be used as delivery vehicles for genetic materials and drug therapy. Exosomes derived from synovial mesenchymal stem cells can reach the chondrocytes and is expected to become a promising carrier for nucleotide drugs to penetrate and target cartilage (Bao and He, 2021). Tao et al. first proposed the use of extracellular vesicles (PDLLA-PEG-PDLLA) as nanoscale carriers for the treatment of OA (Tao et al., 2021). Exosomes derived from synovial mesenchymal stem cells can transmit nucleic acids to chondrocytes to promote chondrocyte proliferation. As sleep has been found to be beneficial to cartilage repair, sleep-related circular RNA (circRNA) cartilage repair was first screened out using melatonin treatment and small extracellular vesicles (sEVs) carrying sleep-related circRNA (circRNA3503) were constructed. Poly (D, L-lactide)-b-polyethylene glycol-b-poly (D, L-lactide) triblock copolymer gel was used as the carrier of sEVs. *In vitro* experiments have shown that this system could promote the regeneration of chondrocytes and reduce the progressive loss of chondrocytes and it is an effective treatment to prevent the progression of OA (Tao et al., 2021). Lipid-based nanocarriers can transport DNA or RNA into cells. These particles are sometimes trapped by endocytosis and the release of nucleic acid structure is limited. To solve this problem, Yan et al. proposed another nanocarrier (HA-coated p5RHH), namely cytolytic peptide, which was modified to reduce its pore-forming ability and retain its ability to be inserted into a bilayer membrane (Yan et al., 2020). The modified peptide forms a self-assembled nanostructure, and after being stabilized by HA, the short interference RNA (siRNA) can be quickly transmitted to cytoplasm and the expression of specific genes can be down-regulated *in vitro* and *in vivo*. The nanocomposite was transmitted to human cartilage explants to antagonize β-catenin/WNT3a signal transduction, leading to the decrease of chondrocyte apoptosis. However, more experiments are needed to verify the validity in the future (Yan et al., 2020).

PTOA is an inflammation of the joints caused by acute injury, followed by progressive degradation of the articular cartilage. Bedingfield et al. loaded NPs with siNPs in poly (lactic acid-glycolic acid copolymer) (PLGA) microporous plates (μPLs) and obtain siNP-μPLs, which allowed siNPs to be retained in joints for a longer time (Bedingfield et al., 2021). MMP13 is up regulated in PTOA and degrades key cartilage structural protein type II collagen. In mouse PTOA model, the treatment with siNP-μPLs against MMP-13 (siMMP-13-μPLs) effectively reduced the expression of MMP-13 gene and the production of MMP-13 protein in joint tissues. It slows down the degeneration of articular cartilage, synovial hyperplasia, osteophyte formation and pro-inflammatory gene expression in PTOA, proving that siNP-μPLs has a good potential for the treatment of PTOA (Bedingfield et al., 2021). Although microRNA gene therapy can delay the progression of PTOA, due to the limitations of the gastrointestinal environment, an effective gene delivery vehicle is needed to deliver oral therapeutic drugs. Zhang et al. proposed the yeast cell wall particle-mediated nanotube-RNA delivery system (NPs-YCWP) as a delivery carrier for the oral route for the treatment of PTOA, YCWP can resist the corrosion of gastrointestinal juice and achieve the purpose of oral administration. NPs-YCWP provide a new idea for the nano delivery systems (Zhang et al., 2020).

## 5 Osteocartilage regeneration scaffold

Scaffolds are the key factors for osteochondral tissue engineering, and it is difficult to prepare suitable scaffolds for osteochondral defects. Osteochondral defects are not identical between individuals. Therefore, individualized treatment is the best option when conditions permit. The scaffold is an endophyte, which needs to have good biocompatibility and synchronous degeneration with cartilage regeneration (Wei and Dai, 2021). Applications of nanotechnology in scaffolds include the addition of NPs to conventional scaffolds to produce nanoscale features, and the direct fabrication of nanofiber scaffolds.

Bone is a nanofiber structure of calcified connective tissue. Nano scaffolds can play a role in supporting, repairing cartilage, and inducing osteogenesis. Currently, cartilage repair research is mostly focused on bone marrow mesenchymal stem cells, however these cells cannot accomplish good repair effects on their own. The combination of scaffolds and bone marrow mesenchymal stem cells may be a more promising strategy, especially 3D printed scaffolds, which may produce more satisfactory results in the application of individualized therapy (KöSe et al., 2018; Su et al., 2021).

Chen et al. reported an HA cross-linked three-dimensional scaffold (3DHAS) consisting of the two-dimensional electrospun poly (l-lactide-co-ε-caprolactone)/silk fibroin scaffolds and HA. The bionic scaffold has good mechanical property and biocompatibility. After being implanted into the rabbit’s full-thickness articular cartilage model for up to 12 weeks, the researchers observed its ability to repair cartilage (Chen et al., 2021). Another acellular HA scaffold provides stromal cell-derived factor 1α, mesenchymal stem cells (MSCs) and TGF-β3 to promote cartilage tissue formation. Notably, the biological activities of these two factors were demonstrated *in vitro*: both stromal cell-derived factor (SDF) and TGF increased cell migration, and TGF increased matrix formation of MSCs. Animal experiments have shown that scaffolds that release both SDF and TGF are less effective than scaffolds that release TGF alone. The reason behind this is unclear. This reminds us that unexpected results may occur when we switch from *in vitro* experiments to *in vivo* experiments (Martin et al., 2021). Bone MSCs promote tissue repair through paracrine under specific environments. Chitosan/polyvinyl alcohol nanofiber scaffolds (CS/PVA scaffolds) can be used to simulate the extracellular matrix and facilitate the proliferation and differentiation of human adipose tissue-derived mesenchymal stem cells into chondrocytes (Nour-Eldeen et al., 2020).

### 6 Nano lubricant

**FIGURE 3:**
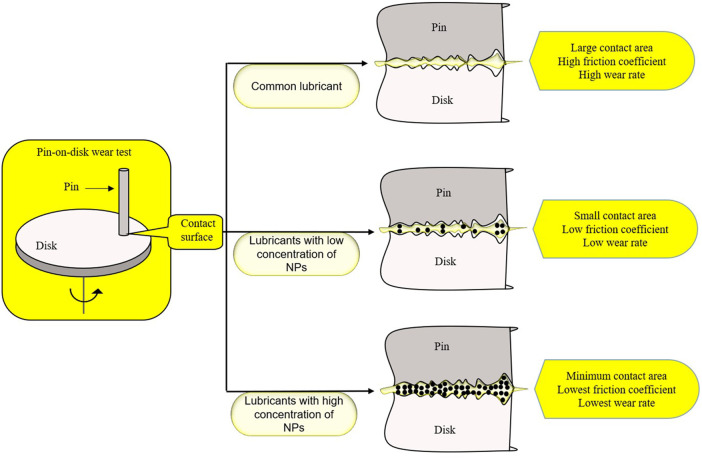
Adding GNP to the lubricant can improve the effect of the lubricant. The nano-GNP in the lubricant reduces the contact area by filling, and the effect of the lubricant can be the best when the concentration of GNP reaches a certain degree.

Human hip and knee articular surfaces are the most efficiently lubricated surfaces known in nature. Under physiological pressure, the joint friction coefficient is as low as 0.001, which is of great importance for the joint health of human body. Therefore, finding a more effective lubricant for OA patients may be of great significance for the improvement of their joint function (Lin and Klein, 2021). The lubricant suspension added with NPs is called nano-lubricant, and NPs can be stably suspended in the lubricant to enhance the lubrication effect of the lubricant (Bakak et al., 2021). Graphite nanoplatelets (GNP) can improve the lubricating property of the lubricant. It was found that there was a critical concentration of GNP for lubrication promotion, at which the promotion will reach a peak (Figure 3). There is a minimum contact area between the objects at the peak, which also indicates that the addition of NPs is more conducive to filling the rough part of the contact surface (Omrani et al., 2021). In recent years, a new lubricant, 1,3,9 malondialdehyde semi-dendritic hyperbranched polyglycerol (Mega HPG), has been proposed. This is a single nanoscale polymer particle. Mega HPG has the advantages of high-water solubility, low intrinsic viscosity, compactness, specific viscosity, and hydration, which can reduce the friction coefficient between the hard and soft surfaces. Mega HPG acts as interposed single molecule ball bearings, reducing the coefficient of friction between hard and soft natural surfaces in a size-dependent manner (Anilkumar et al., 2020).

## 7 Discussion

The development of nanotechnology provides fresh ideas for its application in OA treatment from a variety of perspectives, including the changes in drug delivery methods and the development of new materials, all of which will have a substantial impact on the clinical strategies of OA (Table 1). Some of the nanotechnologies mentioned above have begun to be used into clinical settings, while others are still being researched and developed in laboratories. As drug delivery systems, NPs can deliver medications to specific areas in a direct manner, extend the action time of drugs through physical and chemical mechanisms, improve drug efficacy, and fulfill the goal of increasing treatment efficiency. Although genetic engineering may hold the key to complete solution for OA, it is still in an its infancy. The gradually identified gene therapy targets, as well as the maturing nanotechnology, will provide a strong foundation for future gene therapy development. Nanoscale scaffolds offer improved mechanical characteristics, histocompatibility, and cartilage regeneration ability. Currently, lubricants are the most common application of nanotechnology in OA, but there are additional possibilities in the future.

**TABLE 1:** Summary of the research work of nanotechnology in the treatment of osteoarthritis.

	NPs	Advantages	Example	References
Drug delivery system	Chitosan	Rich in amino groups	Chitosan	Kang et al. (2014)
		Promote cartilage formation	Polycationic chitosan	
	Aliphatic polyesters	Biocompatibility and biodegradability	Polylactic acid	Salama et al. (2020)
			Polyglycolide	
	Targeting NPs	Active targeting	HA-NPs	Kang et al. (2021), Rey-Rico et al. (2017), Geiger et al. (2018)
		Resistance to hyaluronidase digestion	IL-1RA	
			Dendrimers	
	Stimulate reactive NPs	Heat response	Hollow dextran/poly (N-isopropylacrylamide) NPs	Song et al. (2021), Lachowicz et al. (2020), Deloney et al. (2020), Kang et al. (2016), Zhao Y. et al. (2019)
		Large drug load	Chitosan-modified molybdenum disulfide nanosheets	
		Double release	pNIPAm	
		Vitro control		
	Micelles and liposomes	Amphiphilic	Hydrogen peroxide-sensitive nanomicelle	Wu et al. (2021), Lan et al. (2020); Bhatia et al. (2020)
		Good plasticity	Liposomal gel	
Gene delivery system	Exosomes	Penetrate and target cartilage	Exosomes derived from synovial mesenchymal stem cells	Tao et al. (2021)
	Cytolytic peptide	The pore-forming ability is reduced, and the ability to insert bilayer film is retained	Modified peptide	Yan et al. (2020)
	siNP-μPLs	Long-term retention	siMMP13-μPLs	Bedingfield et al. (2021)
	Yeast cell wall particle	Oral administration	Yeast cell wall particle-mediated nanotube-RNA delivery system	Zhang et al. (2020)
Scaffold	Bionic scaffold	Good mechanical property and biocompatibility	HA cross-linked three-dimensional scaffold	Wei and Dai, (2021)
	Nanofiber scaffolds	simulate the extracellular matrix	Chitosan/polyvinyl alcohol nanofiber scaffolds	Nour-Eldeen et al. (2020)
Lubricant	Additive NPs	Improve the lubricating property of the lubricant	GNP	Omrani et al. (2021)
	Nanoscale polymer particle	High-water solubility	Mega HPG	Anilkumar et al. (2020)
		Low intrinsic viscosity		
		Compactness		
		Specific viscosity and hydration		

However, nanotechnology is costly and unfamiliar to most OA patients, limiting its therapeutic use. This also implies that researchers must continue to make unwavering attempts to address the above-mentioned flaws as much as possible. There are still many shackles that need to be broken in the application of nanotechnology in OA treatment. For example, the nano drug delivery system needs to improve efficiency and realize the transformation from intra-articular injection to oral drugs and then to external drugs; to achieve ultra-early treatment of OA, we need to block the progress of OA from the source (gene); the implant material needs improvement to accelerate bone healing or extend the service life of the joint replacement prostheses. Furthermore, in the face of surgical infection, it is vital to look for better nanotechnology-based remedies. Internal plants covered with nano-sized hydroxyapatite have been put into clinical use, and 3D printing for bone defect repair and degradable internal fixing is in the emerging and booming stage.

The emergence of CRISPR/Cas9 technology promotes the rapid development of genetic engineering. At present, the research on gene editing and osteoarthritis treatment is being carried out step by step, compared with other gene therapy, gene editing has the advantages of thoroughness and persistence, but safety is still a problem to be solved. CRISPR/Cas9 can be used more safely and efficiently in clinic with the help of nano-delivery system (Zhao L. et al., 2019; Hussain et al., 2022). The emergence of new therapeutic procedures increases the difficulty in selecting clinical treatment alternatives, requiring doctors to choose the optimal plan according to the patients’ condition more carefully. Nanotherapy will be further developed, and step-by-step therapy programs will be formulated, but this requires future multidisciplinary collaboration.
